# Universal duties in a fragmented world: why Europe must reclaim Kantian ethics for global health governance

**DOI:** 10.1093/eurpub/ckag019

**Published:** 2026-02-06

**Authors:** Martin McKee, Tiago Correia

**Affiliations:** Department of Health Services Research & Policy, London School of Hygiene & Tropical Medicine, London, United Kingdom; Associate Laboratory in Translation and Innovation Towards Global Health,(LA‐REAL), Global Health and Tropical Medicine, Institut de Higiene e Medicina Tropical, Universidade Nova de Lisboa, Lisbon, Portugal

Long-standing norms that have safeguarded global health are being discarded, threatening the foundations of cooperation and equity on which health security depends. Health facilities, health workers, and humanitarian corridors are being attacked deliberately in many conflict settings. Mechanisms intended to safeguard collective health, from guarantees of humanitarian access to pandemic preparedness frameworks, are being eroded by unilateral actions, such as the US withdrawal from global bodies, and a disregard for international law. The consequences are not abstract: they include delayed outbreak detection, denial of access to vaccines and essential medicines, and the normalisation of assaults on the protections on which global health depends. These developments raise a fundamental question for the global health community: can collective health be protected when political action no longer accepts universal obligations?

In such times of profound uncertainty, it helps to return to first principles. An obvious source is the 18th-century German philosopher Immanuel Kant, who argued that actions should rest on universal moral duties rather than contingent preferences. At its core, he demands that we act only on maxims we would want to have as universal law and treat humanity always as an end, never merely as a means.

These ideas appear in his 1785 Metaphysics of Morals, where he presents what he calls the categorical imperative in several mutually reinforcing formulations [1]. His universal law principle states that a rule is moral only if it can be applied by everyone without contradiction. Policies must be defensible as universal standards, not just convenient for some. His principle of humanity insists that every person be treated as an end in themselves, never merely as a means, affirming the intrinsic worth of all individuals. Together, these demand consistency and respect for human dignity across borders and populations. Another of Kant’s ideas, the kingdom of ends, a community of rational agents legislating universal laws, maps naturally onto multilateral health governance. Kant’s later political writings reinforce this view. In Perpetual Peace (1795), he argues for lawful relations between states, anticipating norms that underpin the protection of all individuals irrespective of nationality [2].

Kantian ethics views global cooperation as a categorical duty. As such, we should establish arrangements that any rational agent would want to see applied universally, and that honour every person as an end in themselves. These arrangements require binding commitments to equity, reciprocity, and shared responsibility, operating on principles of justice rather than political expediency.

What do these principles mean in the field of global health? Kant’s first formulation requires that states do something only if they are willing to see it applied to everyone. If they reject this, acting only in their own interests, global health cooperation would become impossible.

Systems, such as those enabling disease surveillance, vaccine sharing, or emergency response, depend on reciprocity. A world in which states hoard resources and disregard collective agreements cannot manage pandemics or transnational health threats. This was evident during COVID-19: initiatives such as COVAX, designed for equitable vaccine distribution, faltered when high-income countries pursued bilateral deals and stockpiled doses [3]. Similarly, ongoing Pandemic Treaty negotiations, intended to create binding commitments on preparedness and equity, face resistance from states unwilling to limit sovereignty, underscoring the fragility of global solidarity [4].

The second formulation, the principle of humanity, requires that we treat every person as an end in themselves, never merely as a means. Policies that subordinate global health to domestic advantage or use aid as leverage instrumentalise human lives.

Under Kantian ethics, such stances are morally indefensible. They erode the trust and cooperation essential for health security and justice. By contrast, multilateral engagement, through mechanisms such as COVAX and the Pandemic Treaty, reflects duties of beneficence and respect, operationalising maxims that could be universally adopted. The moral imperative is clear: global health governance must rest on principles of universality and dignity, not contingent calculations of power.

In a world of interdependence, Kant’s categorical imperative demands that nations act not as isolated actors but as members of a “kingdom of ends,” committed to laws that uphold the equal worth of all persons. The core elements of global health, including surveillance, preparedness, and the protection of health workers, rely on rules that apply regardless of politics. When exceptions occur, the result is moral incoherence and system breakdown.

Kant’s imperative offers a foundation of universality and respect for persons. Europe bears a special responsibility here because its 20th-century history demonstrates the catastrophic consequences of abandoning universal norms [5]. The collapse of multilateralism, the rise of nationalism, and the disregard for human dignity led to war and genocide on an unprecedented scale. Out of that devastation emerged institutions and treaties grounded in the ideals Kant articulated: universality, reciprocity, and respect for persons. Yet their promise remained partial, securing dignity for some while failing to prevent violence and injustice elsewhere, particularly in the Global South. To neglect these principles now will betray that legacy and risk repeating the failures that once shattered the continent and the world.

Conflict of interest: None declared.

## Data Availability

Not applicable.
